# The Role of Gene Expression in Stress Urinary Incontinence: An Integrative Review of Evidence

**DOI:** 10.3390/medicina59040700

**Published:** 2023-04-03

**Authors:** Iva Miličić, Mislav Mikuš, Adam Vrbanić, Držislav Kalafatić

**Affiliations:** 1 Department of Gynecology and Obstetrics, University Hospital Centre, 10 000 Zagreb, Croatia; 2 Medical School, University of Zagreb, 10 000 Zagreb, Croatia

**Keywords:** stress urinary incontinence, gene expression

## Abstract

Stress urinary incontinence (SUI) is defined as unintentional urine leakage occurring as a consequence of increased intraabdominal pressure due to absent or weak musculus detrusor contractility. It affects postmenopausal women more often than premenopausal and is associated with quality of life (QoL) deterioration. The complex SUI etiology is generally perceived as multifactorial; however, the overall impact of environmental and genetic influences is deficiently understood. In this research report, we have disclosed the upregulation of 15 genes and the downregulation of 2 genes in the genetic etiology of SUI according to the accessible scientific literature. The analytical methods used for the analysis of gene expression in the studies investigated were immunohistochemistry, immunofluorescence staining, PCR, and Western blot. In order to facilitate the interpretation of the results, we have used GeneMania, a potent software which describes genetic expression, co-expression, co-localization, and protein domain similarity. The importance of this review on the genetic pathophysiology of SUI lies in determining susceptibility for targeted genetic therapy, detecting clinical biomarkers, and other possible therapeutic advances. The prevention of SUI with the timely recognition of genetic factors may be important for avoiding invasive operative urogynecological methods.

## 1. Introduction

Stress urinary incontinence (SUI) is defined as unintentional urine leakage occurring as a consequence of increased intraabdominal pressure due to absent or weak musculus detrusor contractility [1]. The evaluation of the pervasiveness significantly diverges in the published literature, which is due to altering nomenclature, different diagnostic approaches, and the population samples examined [2,3,4]. The available data disclose that SUI affects up to 14% of premenopausal and up to 35% of postmenopausal women [5], unfavorably impacting quality of life (QoL), sexual satisfaction, and overall psychological prosperities [6,7]. Although the gravity of the diagnose may be perceived as light, it does carry a substantial economic burden. Based on data from the multicentric study, Papanicolaou et al. suggested that the total cost of SUI is approximately GBP 818 million in the UK annually [8]. Pathophysiologically, the prevailing theory of SUI occurrence is implicating deficient urethral function and the impairment of supportive tissue, such as the levator ani muscle, endopelvic sheet, and pubourethral ligament [9].

Moreover, the large impact of surrounding intraabdominal pressure on SUI has been extensively researched.

For instance, the Norwegian EPINCONT study described a correlation between increased BMI and severe urinary incontinence [10]. Compared to women with a BMI less than 25 kg/m^2^, the odds ratio (OR) for the following BMI groups was from 2.0 (BMI 25–29 kg/m^2^ group) to 5.0 (BMI 40 kg/m^2^ or more) [10].

The complex SUI etiology is generally perceived as multifactorial; however, the overall impact of environmental and genetic influences is deficiently understood. Factors such as age, childbirth, and pregnancy are known to increase the incidence of SUI [10]. Additionally, one study found a two-fold risk increase in developing urinary incontinence in women who were current or former smokers compared to the non-smoking group [10].

Numerous epidemiologic surveys have found hereditable predisposition to SUI [11,12,13,14], although hindered by a rather small number of women studied and recruitment bias. One published study used the widespread Swedish Twin Register to estimate the relative importance of genetic and epigenetic factors on the accountability of SUI [15]. In their research, Altman et al. identified 3376 complete monozygotic and 5067 dizygotic same-sex female twin pairs. In terms of SUI, genomic factors contributed about 40% in liability [15].

Regarding the genomic mechanisms of SUI development, currently published studies have emphasized alterations in extracellular matrix proteins and single nucleotide polymorphisms in candidate genes [16,17]. Although the SUI genetic susceptibility is widely accepted, further supportive evidence to these theories is needed. Therefore, this review aims to summarize the data available in the literature regarding the role of gene expression in SUI development.

## 2. Materials and Methods

### 2.1. Study Design

For the purpose of this integrative review, research was performed to identify publications questioning the genetic and the non-environmental contribution to the development of SUI comparative to healthy women. The research was conducted in accordance with the Preferred Reporting Items for Systematic Reviews and Meta-Analyses (PRISMA) recommendations [18]. Taking into consideration the essence of this research, Institutional Review Board permission was not relevant.

### 2.2. Eligibility Criteria

All included studies were obligated to incorporate the results from the gene expression analysis and analysis type. In this study, candidate genes were considered related to SUI if their expression was consistently validated across all the statistical and analytical methods.

The inclusion criteria applied in this integrative review were as follows:

1. Gene expression analysis of SUI women relative to healthy women;
2. Experiments on samples from human tissue;
3. Gene expression detection and quantification using molecular techniques of polymerase chain reaction (PCR), immunohistochemistry, Western blot, and immunofluorescence staining;
4. Articles written in the English language.

We excluded patients with POP-Q ≥stage III and type III SUI based on Blaivas clinical classification. Moreover, studies of women with prior pelvic surgery and pelvic radiotherapy, neurological disorders (e.g., epilepsy, Parkinson disease, multiple sclerosis, cerebrovascular insult), endometriosis, and connective tissue disorders were excluded.

The eligibility criteria were applied to the selected articles with the support of the reference manager software Endnote™ Version 1.19.4, by which duplicates were identified and excluded.

### 2.3. Information Sources and Search Strategies

A literature search was conducted through the PubMed and EMBASE electronic databases, focusing on distinguishing articles published in English between 1 January 1990 and 31 August 2021. Two reviewers (D.K. and M.M.) performed an independent search of the sources. The search was performed using MeSH terms and text words: “urinary incontinence”, “stress urinary incontinence”, “urination disorders”, “voiding dysfunction”, “involuntary urination”, “genetics”, “genetic predisposition”, “matrix metalloproteinase”, “collagen”, “collagen mutation”, “gene expression”, “clinical trial”, and “randomised clinical trial”. Additionally, the Boolean operators “AND” and “OR” were applied for “stress urinary incontinence” and the other terms, respectively.

### 2.4. Study Selection and Data Extraction

**Figure 1:**
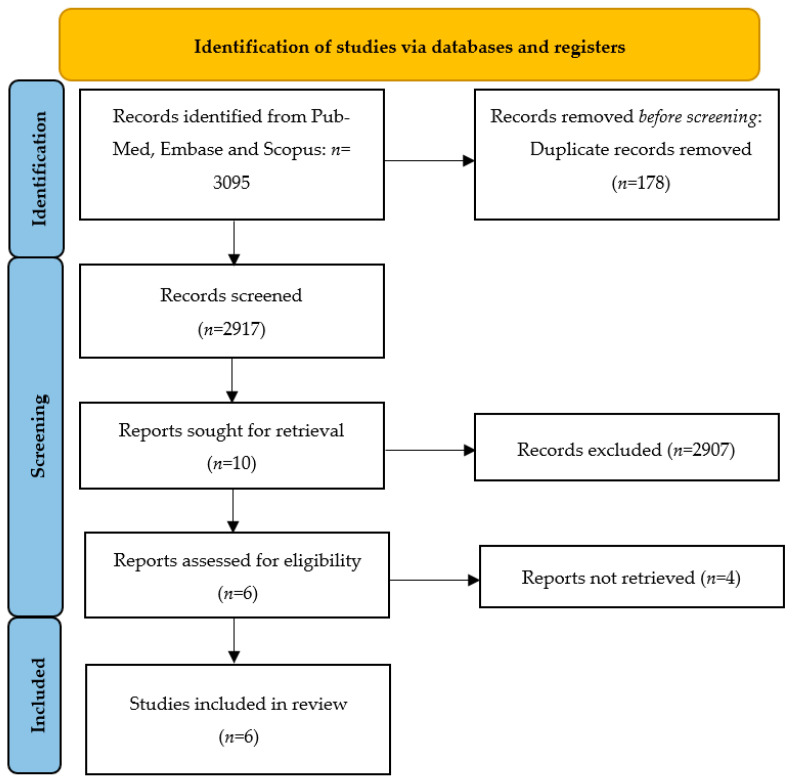
The Preferred Reporting Items for Systematic Reviews and Meta-Analyses (PRISMA): manuscript selection strategy and flow diagram.

Titles and abstracts were independently screened by two authors (D.K. and M.M.). The study selection performed by two independent reviewers reduced the chance of excluding relevant studies. The same authors independently assessed studies for inclusion and extracted data regarding study features, populations, and outcomes. A manual search of the references of included studies was also performed to avoid missing relevant data. Any disagreement or uncertainty were resolved through discussions among the researchers until a consensus was achieved. Narrative or systematic reviews, case reports/case series, and conference abstracts were excluded from the analysis. Figure 1 depicts the overall manuscript selection strategy and flow diagram.

### 2.5. Data Items

**Figure 2:**
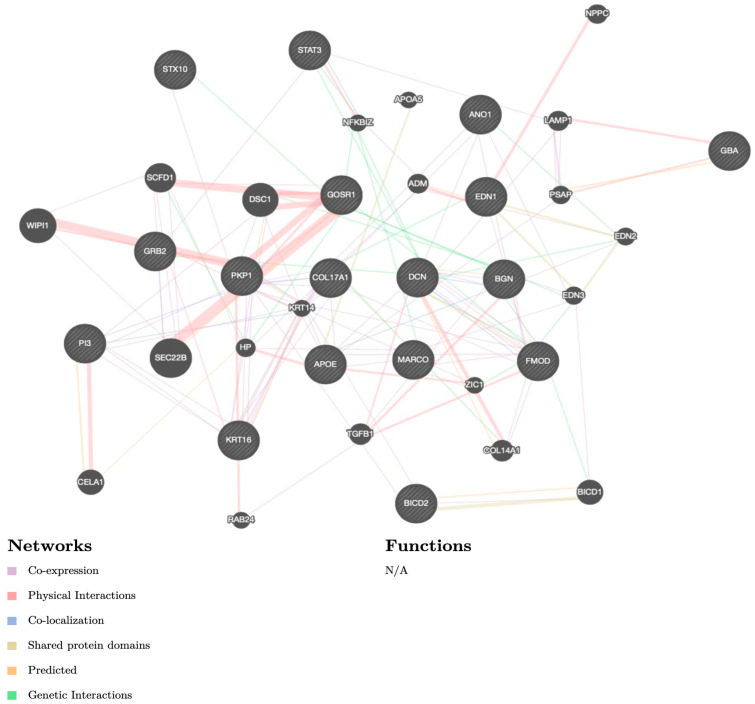
GeneMania software diagram. The color of the line attaching the genes explains the kind of interaction: co-expression in purple lines, physical interactions in red lines, co-localization in blue lines, shared protein domains in bright yellow lines, predicted communication in orange color, and genetic interactions in green lines. **Note:** N/A—not available.

Every article was meticulously read and the information on the research theme, methods, results, and authors was recorded in Table 1 and Table 2. In Table 1 and Table 2 are the recorded study types and main findings from the articles. In addition to the primary outcomes, a list of 17 identified genes were applied to GeneMania software (www.genemania.org (accessed on 10 November 2022)) in order to show co-expression, physical interactions, co-localization, shared protein domains, predicted interactions, and genetic interactions (Figure 2). The rationale of GeneMania was to use the results for the future prioritizing of gene candidates for future functional assays. The color of the line attaching the genes explains the kind of interaction. The diagram encloses co-expression in purple lines, physical interactions in red lines, co-localization in blue lines, shared protein domains in bright yellow lines, predicted communication in an orange color, and genetic interactions in green lines.

### 2.6. Risk of Bias in Individual Studies

The methodological quality of the studies was independently assessed by two investigators using the nine-star Newcastle–Ottawa scale (NOS), as presented in Table 1 [19]. Each paper was assessed based on eight features and was categorized into three comprehensive groups, including selection, comparability, and outcome for cohort studies or exposure for case–control studies. We classified studies with a score of 7 or greater as high-quality. The resolution of discrepancies was achieved by a discussion or through consultation with a third investigator (I.M.).

**Table 1:** Newcastle–Ottawa scale (NOS) assessment of included studies.

Author, Year	NOS Score (Selection + Comparability + Outcome)
Chen, 2006 [20]	8
Wen, 2007 [21]	7
Tong, 2010 [17]	8
Liu, 2014 [22]	8
Chen, 2020 [23]	7
Cartwright, 2021 [24]	8

## 3. Results

### 3.1. Study Selection

The electronic searches provided a total of 3095 citations, but after the removal of 178 duplicate records, 2917 citations remained. Of these, 2907 records were excluded after title/abstract screening (not relevant to the review). We examined the full text of 10 publications remaining and, of these, we excluded 4 papers (due to non-English language use and inclusion/exclusion criteria not being applied).

Finally, six manuscripts the met eligibility criteria for the integrative review. A study flowchart summarizing the literature identification and selection is provided in Figure 1.

### 3.2. Study Characteristics and Results of Individual Studies

Table 2 and Table 3 outline the main characteristics of the included publications: study design, study size and population, tissue analyzed, analytical methods used, and result synthesis and interpretation.

The tables display the results of the selected studies reporting the SUI-related genes which were either over- or under-expressed in SUI patients versus continent women. A total of 17 genes were identified to be up- or downregulated in SUI patients [17,20,21,22,23,24]. The analytical methods used for gene expression in the included studies were immunohistochemistry, immunofluorescence staining, PCR, and Western blot.

**Table 2:** General characteristics of studies.

Author, Year	Study Design	Study Population	Tissue Analyzed	Analytical Methods Used	Results Summary
Chen, 2006	Case control	*n* = 26 women; 14 cases and 12 controls	Vaginal wall	Immunofluorescence cell staining, microarray data analysis, PCR, Western blot	SKALP, KRT16, COL17A1, PKP1 were perpetually classified as upregulated genes by both MAS 5.0 and RMA when assessed through analytical methods and as discussed in the study.
Wen, 2007	Case control	*n* = 62 women; 31 cases and 31 controls	Vaginal wall	Immunofluorescence cell staining, PCR, Western blot	BGN, DCN, and FMOD were found to be placed in vaginal tissue along with connective matter (via immunofluorescence cell staining). DCN mRNA was 3-fold higher in the case group in the proliferative phase and 8 times higher in the secretory phase. FMOD mRNA was 2.5 times lower in the case group in the proliferative phase. BGN showed no difference in both phases. Via Western blot: BGN and DCN showed higher density in the case group in the secretory phase, while FMOD showed lower density in the case group in the secretory phase.
Tong, 2010	Case control	*n* = 17 women; 9 cases and 8 controls	Vaginal wall	Microarray data analysis, PCR, Western blot	The four most relevant pathways identified were: SNARE containing STX10, GOSR1 genes Nerve degenerating pathway via GRB2, APOE genes Inositol functioning including GBA gene (APOE, GRB2, GOSR1, and GBA were selected)
Liu, 2014	Case control	*n* = 26 women; 13 cases and 13 controls	Vaginal wall	miRNA microarray data analysis, PCR, Western blot	12 miRNAs were differentially expressed (*p* < 0.05) (5 upregulated, 7 downregulated). The differential expression of these 12 miRNAs predicated 3 miRNA-mRNA pairs for BICD2, GRB2, and STAT3 genes
Chen, 2020	Case control	N/A; Samples were obtained from prostate hyperplasia surgeries in men and bladder outlet obstruction and bladder neck sclerosis surgeries in women.	Urethra	Immunofluorescence cell staining, PCR, Western blot	The upregulation of ANO1 in urethral smooth muscle cells results in doubling the expression in women and female mice compared to cells from men and male mice.
Cartwright, 2021	Case control	*n* = 8979 women; genome-wide association study in 3 independent discovery cohorts of European women.	Urinary bladder	Microarray analysis, RT-PCR	The authors classified two genetic variants associated with SUI. The first, rs138724718, is located near MARCO, functioning as a host protection. The second, rs34998271, is positioned near EDN1, a major smooth muscle contractor.

**Table 3:** Functions of identified and investigated genes involved in stress urinary incontinence.

Full Name	Gene Symbol	Functions
Anoctamin 1	ANO1	- Anion transport through the epithelium and smooth muscle contraction. - Required for the normal functioning of the interstitial cells of Cajal. - Important contributor to chloride transmission in respiratory epithelium.
Apolipoprotein E	APOE	- Contributes to lipid transfer between organs through the plasma and fluid in the interstitium. - Macromolecular and protein–lipid complex remodeling.
Biglycan	BGN	- Contributes to bone development, muscle formation and regeneration, as well as collagen congregation in various tissues.
Protein bicaudal D homolog 2	BICD2	- Microtubule-based movement (transformation of dynein from a non-processive to a highly processive apparatus through dynactin assistance).
Collagen type XVII alpha 1 chain	COL17A1	- Structural component of hemidesmosomes. - May be important in the attachment of keratinocytes to the basal membrane.
Decorin	DCN	- Regulates the amount of fibrils formation. - Role in glycosaminoglycan catabolic process.
Endothelin 1	EDN1	- Vasoconstriction. - Role in tumorigenesis.
Fibromodulin	FMOD	- Contributes to the construction of ECM (regulates the amount of fibrils formation, suspected to play a major part in collagen fibrils genesis).
Glucosylceramidase beta	GBA	- Helps to decompose complex lipids and important for the reparation of membranes.
Golgi SNAP receptor complex member 1	GOSR1	- Conducts transfer from the ER to the Golgi vesicle and intra-Golgi transfer. - Preserves neurons from H2O2 cytotoxic effect.
Growth-factor-receptor-bound protein 2	GRB2	- Yields linkage between cell surface growth factor receptors and the Ras protein signaling pathway. - Fibroblast growth factor receptor.
Keratin 16	KRT16	- Regulates immuno-resistance to skin barrier rift. - Epidermis development.
Macrophage receptor with collagenous structure	MARCO	- Seizes non-opsonized molecules by macrophages in the alveoli. - Possesses pattern recognition receptor (PRR) for attachment Gram-positive and Gram-negative bacteria.
Plakophilin 1	PKP1	- May be important for the desmosome development. - Involved in the formation of the epidermis.
Skin-derived, protease inhibitor 3 (peptidase inhibitor 3)	SKALP (PI3)	- Encodes a protein with antibacterial and antifungal features. - May prevent elastase-mediated proteolysis.
Signal transducer and activator of transcription 3	STAT3	- Regulates various gene expression in stimulated cells. - Has a conducting role in cell growth and apoptosis.
Syntaxin 10	STX10	- Enables transport between endosomes and the trans-Golgi network.

### 3.3. Upregulated Genes

#### 3.3.1. Anoctamin 1 (ANO1)

Anoctamin 1 (ANO1) is a Ca^2+^-activated chloride channel with a ubiquitous expression, participating in important physiological functions including smooth muscle contraction, transepithelial ion transport, and the regulation of neuronal excitability and vascular tone [25]. The expression of ANO1 has been described in the urethral smooth muscle cells [24,25]. Chen et al. confirmed a two-fold higher ANO1 expression in single urethral smooth muscle cells from women and female mice compared to men and male mice [24]. The same group have emphasized that ANO1 in urethral smooth muscle cells contributes to sex differences in urethral spontaneous tone [24].

#### 3.3.2. Apolipoprotein E (APOE)

Apolipoprotein E (APOE) is an encoding glycoprotein which is mostly involved in end lipid and lipoprotein metabolism, promoting the clearance of remnants of triglyceride-rich lipoproteins from the circulation into the liver [26]. APOE is also known for promoting nerve regeneration [27]. According to a case–control study by Tong et al., an upregulated APOE gene expression has been associated with the nerve repair process in patients with SUI [17].

#### 3.3.3. Biglycan (BGN)

The biglycan (BGN) gene is located on the X chromosome in humans, encoding one of the major non-collagenous proteins in mineralized tissues, originally identified in the mineral compartment of bone [28]. According to the published evidence, there is a significant amount of information on the role of BGN on the organization of the extracellular matrix and cell signaling [29]. A depleted extracellular matrix organization could explain the background of BGN upregulation in SUI development [21]; however, further investigations are required.

#### 3.3.4. Protein Bicaudal D Homolog 2 (BICD2)

The bicaudal D (BICD) proteins are adaptor proteins regulating the dynein–dynactin motor complex at various cellular mechanisms, facilitating the trafficking of key cellular cargos [30]. BICD2 mutation has been extensively researched and linked with spinal muscular atrophy type 2 and juvenile amyotrophic lateral sclerosis [30,31]. Liu et al. described BICD2 overexpression in 13 women with SUI compared to controls using miRNA microarray data analysis [22].

#### 3.3.5. Collagen Type XVII Alpha 1 Chain (COL17A1)

Collagen type XVII alpha 1 chain (COL17A1) is a collagenous transmembrane protein and a vital component of mature type I hemidesmosomes [32]. COL17A1 is known to be overexpressed in many malignancies, such as pancreatic adenocarcinoma, and promoting cell multiplication and cell apoptosis inhibition, by activating the NF-κB pathway, may have a role in cancer development and progression [33]. COL17A1 upregulation has been demonstrated in women with SUI compared to continent women, suggesting the importance of the extracellular matrix remodeling pattern in the overall genetic predisposition to SUI [20].

#### 3.3.6. Decorin (DCN)

Decorin (DCN) is a soluble molecule localized in the extracellular matrix of vaginal wall tissue alongside collagen fibrils, thus maintaining the structural integrity of the vaginal wall and adjacent structures [34]. Due to its non-structural features, DCN also reflects the role of a ligand for receptors such as the epidermal growth factor and insulin-like growth factor receptor [34,35]. It also plays a role in immune disorders, calcium homeostasis, fetal membrane signaling, and facilitates wound healing and angiogenesis [34]. DCN overexpression has been implicated in a study by Wen et al. on 31 women with SUI compared to continent women [21]. A higher expression of DCN in tissues from women with SUI could present a causal connection by the degradation of the collagen and elastin fibers of the vaginal wall.

#### 3.3.7. Endothelin 1 (EDN1)

There is growing evidence supporting the role of the endothelin 1 (EDN) axis in female reproductive disorders [36], such as endometriosis, preeclampsia, and ovarian and cervical cancer [37]. A recently published genome-wide association study in three independent discovery cohorts of 8979 European women identified a genetic variant situated near EDN1 and strongly associated with urinary incontinence [23]. This is in line with the previous findings of EDN expression in detrusor smooth muscle and urothelium [38]. Another role of EDN1 in the female reproductive tract has been extensively researched in the setting of a potential therapeutic target for the treatment of bladder overactivity, although with various success [39,40,41].

#### 3.3.8. Golgi SNAP Receptor Complex Member 1 (GOSR1)

Although the upregulated expression of GOSR1 in women with SUI has been demonstrated [17], the main determinant of this Golgi vesicle trafficking protein is neurotransmitter transmission among the soluble NSF attachment protein receptor (SNARE) complex [42].

#### 3.3.9. Growth-Factor-Receptor-Bound Protein 2 (GRB2)

Growth-factor-receptor-bound protein 2 (GRB2) is an intracellular adapter protein involved in catalytic processes, cellular growth, and differentiation [43]. Furthermore, GRB2 has similar functions as GOSR1, significantly contributing to vesicle trafficking and handling toxic protein overload [44]. Two studies identified GRB2 upregulation in women with SUI compared to continent women, implicating the role of GRB2 in vaginal nerve disintegrity in patients who are prone to SUI development [17,22].

#### 3.3.10. Keratin 16 (KRT16)

Keratins presents one of the principal components of the epithelial cytoskeleton. KRT16 encodes acidic type I keratin which de novo transcription is rapidly induced to enhance wound healing [45]. Studies using null mouse models have established the role of KRT16 in maintaining cell adhesion and optimal cell migration during skin wounding [46]. A different KRT16 expression is also associated with psoriasis development [45]. KRT16 upregulation has been confirmed by various methods in women with SUI compared to continent women [20].

#### 3.3.11. Macrophage Receptor with Collagenous Structure (MARCO)

MARCO protein is a plasma membrane receptor located on the macrophage’s membrane, where it identifies pathological molecular patterns and environmental or un-opsonized particles [47]. A very recent prospective study by Savino et al. on 116 infants with a 5-year follow-up has confirmed a correlation between MARCO rs1318645 polymorphisms and susceptibility to repetitive periodic wheezing in children [48]. On the other hand, MARCO rs138724718 polymorphisms have been strongly associated with urinary incontinence, as found by Cartwright et al. using microarray analysis and RT-PCR [23].

#### 3.3.12. Plakophilin 1 (PKP1)

Plakophilins are proteins which are known to be essential for desmosomal adhesion [49].

Desmosomes are cell-junctions localized in epithelial tissues, specifically the ones which are regularly undergoing mechanical stress [49]. PKP1 is proven to intensify desmosome arrangement by enlarging the size and number of desmosomes in vitro and in vivo [50]. Alterations in PKP1 are present in patients with skin fragility syndrome [50,51] and because of its tumor-suppressing role, is inhibited in prostate cancer [50,51]. PKP1 is found to be over-expressed in women with SUI in comparison to continent women [51].

#### 3.3.13. Skin-Derived Antileukoproteinase (SKALP/PI3)

Skin-derived antileukoproteinase is also known as the elafin gene. It is a potent inhibitor of human leukocyte elastase and proteinase 3. SKALP expression is linked to pathological skin lesions; it is not expressed in the healthy epidermis [50,51,52,53,54]. Chen et al. stated that the upregulation of the elafin gene is also associated with SUI [20] using Western blot, IF cell staining, and competitive quantitative PCR for gene over-expression confirmation.

#### 3.3.14. Signal Transducer and Activator of Transcription 3 (STAT3)

The signal transducer and activator (STAT3) is known to play a key role in the regulation of tumor response [55]. Research on cancer therapy indicates targeting STAT3 as a potentially important curative approach toward malignancies [55]. Liu et al. concluded that STAT3 upregulation manifests SUI [22].

#### 3.3.15. Syntaxin 10 (STX10)

Syntaxin 10 is a SNARE (SNAP receptor) protein which is included in trans-Golgi vesicular complex transport. SNAP receptors are N-ethylmaleimide-sensitive factor attachment proteins.

According to Tong et al., the upregulation of the Golgi SNAP receptor complex initiates a neurodegenerative pathway in SUI manifestation [17].

### 3.4. Downregulated Genes

#### 3.4.1. Fibromodulin (FMOD)

Fibromodulin is one of the small proteoglycans composing the extra-cellular matrix (ECM) of vaginal tissue [21]. It is mainly produced by collagen, except the FMOD, vaginal tissue ECM, is composed of elastin, collagen, and other large and small proteoglycans such as biglycan (BGN) and decorin (DCN) [21]. The ECM plays an important role in the mechanical function of supportive tissue; ligaments; and pelvic floor muscles. The disfunction of these structures takes part in the complex pathophysiology of SUI. Wen et al. determined a significantly lower FMOD mRNA expression in women with SUI during the secretory and proliferative phase than in the control groups [21].

#### 3.4.2. Glucosylceramidase Beta (GBA)

Glucocerebrosidase is a gene responsible for the production of a lysosome-active enzyme, beta glucocerebrosidase. Mutations and polymorphisms in the GBA gene have been found in the Gaucher disease [56]. Microarray data analysis by Tong et al. points out the decreased expression of GBA in degenerated pelvic tissue by 0.25-fold in women with SUI compared to healthy control groups [17].

## 4. Discussion

This literature review identified 17 genes as possible predisposing factors for the development of clinically significant manifestations of SUI. The multifactorial and epigenetic pathophysiology has been thoroughly described in the literature to date, but the genetic amplification of SUI remains an under-researched area with a possible great therapeutic potential. In this research report, we have disclosed the upregulation of 15 genes and the downregulation of 2 genes in the genetic etiology of SUI according to the accessible scientific literature. The analytical methods used for the analysis of gene expression in the studies investigated were immunohistochemistry, immunofluorescence staining, PCR, and Western blot. In order to facilitate the interpretation of the results, we used GeneMania, a potent software which describes the genetic expression, co-expression, co-localization, and protein domain similarity. This software was used to determine the linkage and interaction between the prior mentioned genes. The network of biochemical molecules in the ECM of the vaginal tissue forms a support in pelvic muscles and soft tissue. Alterations in the complex and various components of the ECM structure are found to be the result of upregulated or downregulated gene expression. FMOD, BGN, and DCN genes were found to be intertwined in the ECM alongside collagen and elastin [21]. The hormonally dependent downregulation of the FMOD expression in the proliferative phase in contrast to the more potent expression of BGN and DCN proteins in the secretory phase has been described by Wen et al. However, extensive further research on the hormonal dependance of the gene expression is necessary. Chen and colleagues summarized the upregulation of ECM genes using microarray analysis, proving that the expression of genes which encode the function of elastin, such as elafin, COL17A1, KRT16, PKP1, are upregulated in women with SUI [20]. Whilst EDN1 and MARCO genes both help to regulate smooth muscle control, and the upregulation of MARCO has been associated with SUI manifestation [23], research on the specifics of EDN1 targeting SUI pathophysiology are still vague and require an additional scientific approach. Pelvic floor nerve injury and repair mechanisms play a role in the development of SUI and are targeted by the upregulation of APOE and GRB2 and by the downregulation of the expression of the GBA gene, as has been accurately proven by microarray analysis [17]. The same group of authors have also described the linkage between the upregulation of STX10 and GOSR1 genes and pelvic neurodegenerative changes in women with SUI [17]. The BICD2 gene has been linked with various muscular tissue pathologies, and its part in SUI etiology has also been proven [22]; however, a larger amount of examinees may offer a more detailed comprehension. A study by Isaili and colleagues [51] included an earlier review of the genetic regulation of SUI, however, their research presents a total number of 11 genes included in the pathology of SUI. Our unique standpoint of this research includes a systematic review of 15 genes known to date, which are proven to be upregulated or downregulated in SUI combined with their molecular as well as clinical characteristics.

## 5. Conclusions

The importance of this review on the genetic pathophysiology of SUI lies in determining susceptibility for targeted genetic therapy, detecting clinical biomarkers, and other possible therapeutic advances. The prevention of SUI with a timely recognition of the genetic factors may be important for avoiding invasive operative urogynecological methods.

## Data Availability

Not applicable.
